# Smartphone and AI Workflow for 3D Printed Plate for Presurgical Therapy in Cleft Lip and Palate: Retrospective Evaluation of Outcomes

**DOI:** 10.1177/10556656251400877

**Published:** 2025-11-28

**Authors:** Prasad Nalabothu, Hemwati Nandan, Srinivas Gosla Reddy, Andreas A. Mueller

**Affiliations:** 1 Department of Oral and Craniomaxillofacial Surgery, 27209 University Hospital Basel, Basel, Switzerland; 2 Department of Pediatrics Oral Health and Orthodontics, University Center for Dental Medicine UZB, Basel, Basel, Switzerland; 3 Facial and Cranial Anomalies Research Group, 686280 Department of Biomedical Engineering, University of Basel, Basel, Switzerland; 4 Department of Clinical Research, University of Basel, Basel, Switzerland; 5 743742 GSR Institute of Cranio-Maxillofacial and Facial Plastic Surgery, Hyderabad, Telangana, India; 6 Pediatric Oral and Craniomaxillofacial Surgery, 30280 University Children's Hospital Basel, Basel, Switzerland

**Keywords:** smartphone, cleft lip, cleft palate, technological innovations.

## Abstract

**Objective:**

To explore clinical feasibility of smartphone and AI-assisted workflow for presurgical orthopedic therapy in newborns with unilateral cleft lip and palate (UCLP).

**Design:**

Retrospective exploratory cohort study.

**Setting:**

A tertiary craniofacial center.

**Patients:**

Twenty consecutive (13 females, 7 males) with non-syndromic complete UCLP treated 2022–2024.

**Interventions:**

Passive presurgical plates fabricated through smartphone scanning of impressions, AI-assisted automated plate design and 3D printing.

**Main Outcome Measures:**

Clinical outcomes (treatment duration, visits, complications) and morphological changes (anterior cleft width, cleft area, IMT angle, maxillary segment areas).

**Results:**

Therapy began at 10.8 ± 5.7 days, lasted 113.5 ± 15.6 days and required a mean of five visits. Anterior cleft width and cleft area reduced 56.8% and 40.8% with significant maxillary segment growth.

**Conclusions:**

Smartphone and AI assisted workflows showed feasibility of safe, efficient presurgical plate fabrication and therapy, streamlining production and accessibility while reducing procedural burden. The findings remain preliminary and should be interpreted with caution.

## Background

In cleft care, early stage interventions, such as presurgical orthopedic therapy, are critical for improving the anatomy of lip, nose, and palate, which consequently enhances surgical outcomes.^1,2,3,4,5^ Traditionally, plaster casts have been used to create models of newborns’ palates, aiding in the fabrication of presurgical orthopedic devices.^6,7^ However, the conventional impression and plaster pouring techniques are cumbersome, time-consuming, and have long-standing limitations.^8^ Although intraoral scanners have improved safety and precision, their high cost and technical demands restrict widespread adoption, particularly in low and middle-income settings.^9,10^

Advances in digital technology have enabled smartphone-based applications to capture high-resolution images through three-dimensional (3D) scanning.^11^ This technology offers a mobile, contact-free, affordable, and fast method to digitally capture the palatal impression morphology in cleft palate.^11^ When combined with open source software and artificial intelligence (AI), these digital surface scans can be processed to generate accurate digital models and automated presurgical plate designs.^11,12^

Although several groups have described digital approaches, there remains a lack of evidence on clinically validated smartphone based workflows in newborns.^13,14^ Recent work demonstrated the feasibility of smartphone-based cleft palate models and explored the potential role of machine learning in automated plate design.^11^ While their study did not include clinical application, it highlights the technological foundations on which our study builds by providing outcome based evidence in real patient cohort. Digital presurgical orthopedic approaches have also gained attention in the recent literature, particularly in low-resource settings where conventional workflows are often impractical.^15^ Despite this shift towards digital and automated methods, outcome validation in clinical cohorts remain limited, representing the gap addressed by our study.

Cleft treatment is usually provided by multidisciplinary teams with a range of clinical and digital skills. The presented workflow enables classical palatal impression taking, which is robust in variable clinical settings with minimal resources, while also facilitating connection to the fully digital and automated workflow for palatal plate fabrication via smartphone scanning. A previous technical note demonstrated that smartphone-based scans of cleft impressions could be processed with AI to generate digital models, design plates, and fabricate them through 3D printing.^16^ That report confirmed feasibility but did not evaluate whether the workflow produced measurable clinical or morphological improvements.

The present study extends previously published work.^16^ We retrospectively evaluated 20 newborn with unilateral cleft lip and palate (UCLP) who were treated using a smartphone and an AI-based workflow. The objective was to determine whether the approach is both feasible and effective in this exploratory cohort study, by assessing clinical outcomes together with quantitative morphological changes in the palate following therapy. This study was therefore designed to provide scientific validation of the workflow as a potential alternative to conventional presurgical orthopedic methods.

## Materials and Methods

### Study Design

This retrospective observational cohort study was designed and reported in accordance with the STROBE guidelines.^17^ It included newborns with complete unilateral cleft lip and palate (UCLP) treated between January 2022 and December 2024 at a tertiary pediatric craniofacial center. Institutional ethics approval was obtained, and written informed consent was secured from all parents or legal guardians prior to therapy**.**

### Participants

Twenty consecutive newborns (13 females and 7 males) with nonsyndromic, complete UCLP were included. The mean age at initiation of therapy was 10.8 ± 5.7 days (range, 2 −25 days). All newborns commenced therapy within the first three weeks of life and continued until primary cheiloplasty at 3–4 months of age. Exclusion criteria included associated craniofacial anomalies, incomplete therapy before cheiloplasty, or non-compliance with appliance use. All patients meeting the inclusion criteria completed therapy.

### Workflow Summary

**Figure 1.**
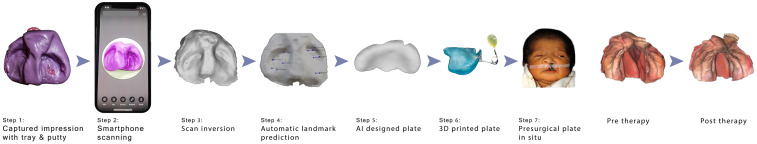
Workflow Illustrating Smartphone-Based Impression Capture, Automated Plate Design, 3D Printing of the Presurgical Plate, Appliance in Place, and Resulting Outcomes of Therapy.

The digital workflow for impression capture, smartphone scanning, AI assisted plate design, and 3D printing followed established methods, as previously described.^16^ In addition, pre and post therapy intraoral scanning (Medit i500, Medit Corp., Seoul, Korea) was performed for 3D evaluation and validation of the workflow with a smartphone. A schematic overview of all steps is provided in Figure 1.

### Therapy Protocol

The presurgical plate was delivered within five hours of impression capture and was to be worn continuously until cheiloplasty. After confirming satisfactory plate fit and retention, the nasal bulb was incorporated on the same day of plate delivery, with minor adjustments made every four weeks during follow-up visits to ensure gentle elevation of alar cartilage, all within the regular visit schedule. The parents were blinded as to whether the plate was fabricated from intraoral scanning or via the spartphone scanning of the impression. Parents were instructed in the insertion and removal of the appliance by use of denture adhesive, once daily cleaning, and monitoring for feeding difficulties or mucosal irritation.^18^ Follow-up visits were scheduled at 4-week intervals to evaluate plate fit, retention, and mucosal health. Adjustments were performed chairside when required, and a replacement plate was fabricated if growth rendered the existing appliance unstable.

### Outcome Measures

Clinical outcomes were obtained from patient records and included therapy duration, the number of visits until cheiloplasty, the frequency of plate adjustments or refabrications, and any complications that occurred, such as plate breakage, pressure sores, airway compromise, aspiration, or feeding difficulties.

Morphological outcomes of the therapy were assessed using three-dimensional (3D) digitised models obtained with the Medit i500 scanner (Medit Corp., Seoul, Korea). These models were analysed using 3-matic (version 15.0, Materialise NV, Leuven, Belgium), with pre-defined anatomical landmarks placed along the alveolar ridges and cleft margins. The evaluated parameters included the anterior cleft width, rotation of the greater segment (IMT angle), true cleft area, and the surface areas of the greater and lesser maxillary segments, all defined according to established landmarks and reference planes in three-dimensional analysi.^18,19^ To ensure reliability, ten randomly selected models were re-measured by two independent observers who were blinded to the treatment stage (baseline or post-therapy). Intra and inter-observer agreement was assessed using intraclass correlation coefficients (ICC).

### Sample Size

All patients who met the inclusion criteria during the study period were enrolled, resulting in a final sample of 20 newborns. As this was an exploratory outcomes study following an earlier feasibility report, no formal sample size calculation was performed. To minimise bias, patients were recruited consecutively and morphological assessments were performed by observers who were unaware of the treatment stage, as well as regarding the input for automated plate fabrication (intraoral scanning or smartphone-based impression scanning). There was no missing data, as all patients completed therapy until the time of cheiloplasty.

### Statistical Analysis

All analyses were performed using R software (version 4.2.2, R Foundation for Statistical Computing, Vienna, Austria), with a significance level of p < 0.05. Continuous variables were first assessed for normality using the Shapiro–Wilk test and summarised as means ± standard deviation (SD). Paired comparisons were made using a paired t-test when the data were normally distributed and a Wilcoxon matched-pairs test when the normality assumption was not met.

## Results

### Clinical Outcomes

A total of 20 newborns (13 female, 7 males) with complete unilateral cleft lip and palate completed presurgical orthopedic therapy using the smartphone and AI-based workflow. The mean age at the initial visit was 10.8 ± 5.7 days (range, 2–25 days). The mean duration of plate therapy was 113.5 ± 15.6 days, extending from initiation until primary cheiloplasty at approximately 3–4 months of age. The mean number of clinical visits per newborn was five (range, 4–7). Initial fitting issues were observed in three newborns, necessitating minor grinding adjustments to the appliance in the labial vestibule or frenulum region.

The overall success of the plate retention procedure was satisfactory, with only two cases (10%) necessitating replacement due to insufficient plate retention. There was no evidence of airway compromise, aspiration events or feeding difficulties. Two newborns experienced minor mucosal irritation, which resolved with simple grinding of the plate. Two newborns required plate replacement due to growth-related retention, but these did not prolong therapy, increase visits, or affect the outcomes. A detailed summary of patient characteristics and clinical outcomes is presented in **(**Appendix, Table 1**).**

### Morphological Outcomes in Palate Following Therapy

**Figure 2.**
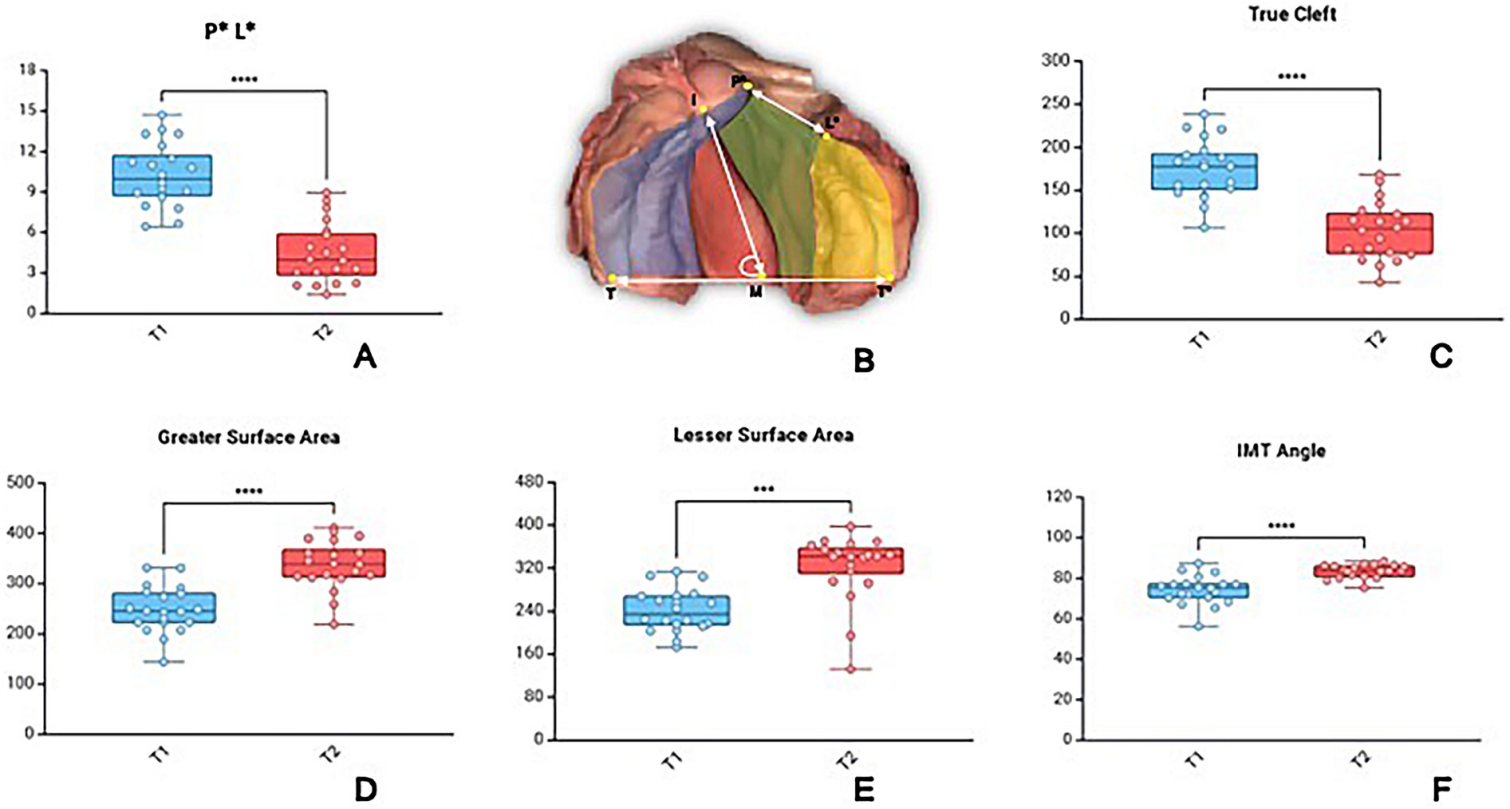
Morphological Changes Following Passive Plate Therapy. (A) Box Plot Showing a Significant Reduction in Anterior Cleft Width (P*L*) (B). Definition of Measured Parameters: P*L* (Anterior Cleft Width), Palatal True Cleft Area (Green Colour), Greater Surface Area (Blue Colour), Lesser Surface Area (Yellow Colour), and IMT Angle. (C) True Cleft Area Reduction, (D) Greater Surface Area Increase, (E) Lesser Surface Area Increase, (F), and IMT Angle Increase. Individual Values for 20 Patients are Represented as Scatter Points Overlaid on the Plots.

Three-dimensional analysis of digitized models demonstrated significant morphological changes over the course of presurgical plate therapy. The anterior cleft width (P*L*) was reduced from 10.27 ± 2.36 mm to 4.44 ± 2.24 mm prior to primary cheiloplasty, corresponding to a 56.8% decrease (Figure 2A). Similarly, the palatal true cleft decreased from 174.23 ± 34.03 mm^2^ to 103.15 ± 33.78 mm^2^ corresponding to a 40.8% reduction (Figure 2C). In parallel, nearly symmetric growth of the maxillary segments was observed, with the greater segment surface area increased by 35.8% (from 248.43 ± 46.36 mm^2^ to 337.37 ± 49.19 mm^2^), while the lesser segment surface area increased by 33.5% (from 241.28 ± 40.09 mm^2^ to 322.35 ± 62.81 mm^2^) (Figure 2D and E).

The IMT angle, reflecting the rotation of the greater segment relative to the intertuberosity line increased significantly from 74.29 ± 7.11° to 83.44 ± 3.37° corresponding to a 12.3% change in angulation (Figure 2F) **(**Appendix, Table 2**)**. The measurement reproducibility was excellent, with both intra- and interrater ICC values consistently above 0.90, which confirms the robust reliability of the morphological analysis. The one outlier of presumable lesser surface reduction in Figure 2E is due to the measurement protocol, which reduces the posterior lesser surface area to a certain extent as it rotates from lateral to medial.

**Figure 3.**
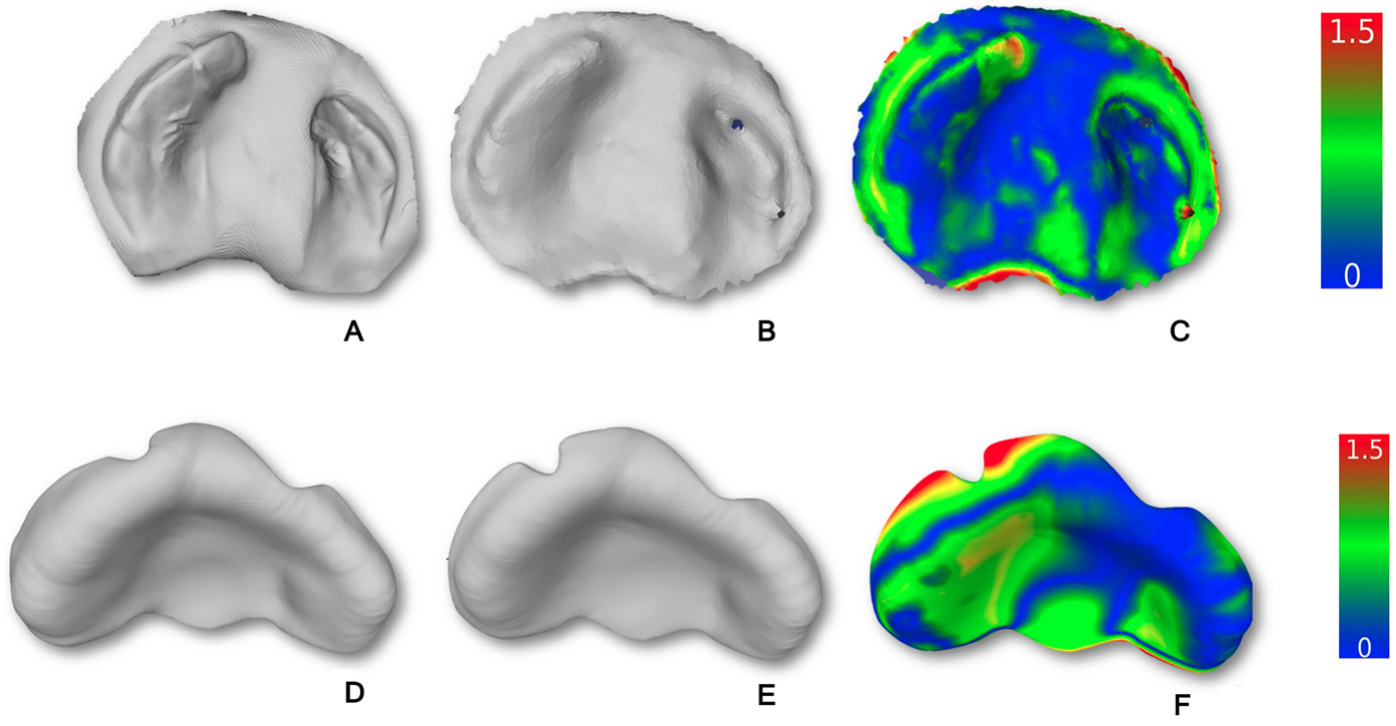
Comparison of the Quality of Impression Scans and Automated Plate Designs Obtained Using an Intraoral Scanner and Smartphone Workflows. (A) Palatal Impression Digitized with an Intraoral Scanner. (B) Palatal Impression Digitized with a Smartphone. (C) Mesh-to-Mesh Distance Histogram (0–1.5 mm) of Palatal Impression Digitized by Intraoral Scanner (A) and Smartphone Scan (B) in CloudCompare. (D) Automated Palatal Plate Computation Based on an Intraoral Scan. (E) Automated Palatal Plate Computation Based on Smartphone Scan. (F) Mesh-to-Mesh Distance Histogram (0–1.5 mm) Comparing Automated Palatal Plate Designs from Intraoral (D) and Smartphone (E) Scans in CloudCompare.

A comparison of scan quality was conducted between using an intraoral scanner Medit i500 scanner (Medit Corp., Seoul, Korea) and a smartphone iPhone 12, Apple Inc., equipped with a two-camera system: a 12-megapixel main and ultra-wide camera, with main ƒ/1.6 aperture, ultra-wide ƒ/1.6 aperture and 120^0^ field of view, featuring 100% Focus Pixels with an open-source app called Scaniverse (^©^ 2024 Niantic, Inc.) to digitize palatal impression. The results revealed RMS (root mean square) values are 0.05 with a mean and standard deviation of 0.35 ± 0.33 mm between meshes for each model (Figure 3 A,B and C). The study also evaluated the accuracy of automated presurgical plate meshes between using an intraoral scanner and a smartphone to digitize the palatal impression. The obtained results showed that RMS was 0.105 and the difference between the two aligned passive plate meshes was around 0.51 ± 0.44 mm (Figure 3 D, E and F)

## Discussion

This exploratory retrospective evaluation supports that a smartphone-based 3D scanning of impression and AI-assisted workflow for presurgical orthopedic therapy in UCLP is clinically feasible and produce meaningful clinical and morphological benefits. By examining twenty consecutive newborns, we demonstrate quantifiable improvements in dimensions of cleft area and maxillary segments. This study extends prior technical work into applied clinical outcomes.

All newborns completed plate therapy successfully until they received their primary surgery, cheiloplasty (around 3–4 months), beginning at a mean age of 10.8 days. The average treatment duration was 113.5 days with approximately 5 scheduled visits from the initiation of treatment till the end of therapy. Minor plate fitting issues were observed in three patients and only two required plate replacement due to instability. Mucosal irritation occurred in two newborns, resolving with minor trimming of the plate at the edges. The nasal bulb ensured comfortable adjustments without any irritation of complications. After an early visit to check hygiene and parental handling of the plate, subsequent visits were not required for the plate itself but were primarily directed towards the nasal bulb. When compared with established presurgical protocols these data suggest a reduced treatment burden.^20,21^ While passive presurgical plates requires minimal mechanical adjustments, the conventional fabrication workflow creates substantial procedural burden through multiple intermediate steps, including plaster pouring, manual trimming, chairside try-ins and adjustments. The proposed smartphone and AI assisted workflow addresses this by: 1) reducing cumulative chairside and laboratory time through automated plate design with precise boundaries and smooth surface finishes, 2) eliminating the need for impression reatkes when plates are lost or damaged trhough digital storage, 3) enabling single visit plate delivery and adjustment scheduling and, 4) standardizing plate parameters to minimize remake due to human error. These workflow efficiencies reduce procedural complexity and handling stress for neonates, caregivers, and clinicians, thereby reducing overall care burden beyond plate adjustments alone. Other techniques require weekly adjustments, translating to 13 −14 visits before primary surgery.^22,23^ In contrast, our workflow provided stable appliances with minimal adjustments, reducing the number of visits to five. This reduction in clinical load has direct implications for families, especially those travelling from far away places and may improve adherence to therapy as well.^22,23^

The morphological analysis of the palate demonstrated significant narrowing of the cleft and favorable growth of both maxillary segments. The anterior cleft width decreased by 56.8% (Figure 2A), while the true cleft area reduced by 40.8% (Figure 2C). The surface area of both the greater and lesser segments increased, confirming that therapy allowed for symmetric growth without signs of growth arrest (Figure 2D and E). The IMT angle also increased, indicating improved orientation of the greater segment (Figure 2F). Such findings are in agreement with earlier reports of presurgical orthopedics which have consistently shown a reduction of true cleft area around 30–40%.^18,19^

### Digital Innovation and Accessibility

This study applies smartphone-based scanning of the impression and automated fabrication of the plate using machine learning methods (ML) in cleft care. The technical feasibility of smartphone-based scanning of cleft palate models and machine learning assisted plate designs has been explored previously.^11^ However their work did not involve clinical application, it provides an important methodological foundation that our present study extends by contributing outcome-based evidence.

While intraoral scanners are now widely adopted in high-resource settings, their purchase and maintenance costs, notebook interface and clinical handling limit broader use.^15^ In contrast, the proposed approach combining smartphone scanning of impression, open source processing, AI-assisted plate design, and 3D printing offers a sustainable and adaptable alternative. Its accessibility makes it particularly relevant for resource limited contexts, while its reproducibility and efficiency demonstrate potential for integration across diverse healthcare systems.

The integration of machine learning for automated plate design represents another innovation.^12^ Automated workflow reduces operator dependency, improves reproducibility, and shortens turnaround time. While smartphone scans are less detailed than intraoral scans, our findings demonstrate that the ML tool can reliably recognize relevant anatomic features, allowing accurate plate fabrication (Figure 3).

### Clinical Implications

The findings of this study support the integration of smartphone and AI-assisted workflows into preoperative cleft care. By enabling the rapid and reproducible fabrication of plates with fewer clinical visits than conventional methods, this approach reduces the burden on families and providers while maintaining effective morphologic adaptation before surgery.^22,23^ As the workflow relies on widely available technologies, it is particularly well-suited to implementation in settings with limited resources, but its efficiency and standardisation also make it applicable in centres with ample resources. Notably, the capacity to store and share digital models fosters multidisciplinary collaboration and facilitates long-term treatment monitoring and outcome assessment.^10^ As digital and machine learning tools continue to advance, this approach provides a practical foundation for scalable and accessible cleft care.

### Learning Curve and Cost Considerations

The smartphone-based scanning and AI-assisted plate design workflow demonstrated a short learning curve and required minimal training. Clinicians already familiar with impression taking could perform scanning with basic instruction, and the AI-assisted plate generation was largely automated, reducing dependence on specialized digital design skills. From a cost perspective, the workflow utilizes readily available devices (a standard smartphone with an open-source scanning application) and open-access software tools, with the only additional expense being 3D printing. These costs are significantly lower compared to commercially licensed CAD platforms and reduce manpower requirements in laboratory fabrication, supporting feasibility even in low-resource clinical settings.

### Limitations of the Study

While this exploratory study provides important early evidence, its retrospective design, small sample size, and lack of a control group limit the strength of generalizability. Furthermore, outcomes were assessed only until cheiloplasty, and longer-term effects of surgical and craniofacial growth remain to be determined. Although smartphone-based scanning demonstrated clinical utility, future studies should also explore and compare automated plate design using intraoral scanner-derived 3D data to benchmark performance across acquisition methods. Prospective, multicenter validation in diverse settings will be important to establish the scalability and broader applicability of this workflow.

## Conclusion

This exploratory study demonstrates the clinical feasibility and workflow efficiency of a smartphone and AI-assisted approach for passive presurgical plate fabrication, offering a practical alternative rather than a replacement for conventional methods. While the clinical outcomes were comparable to those of traditional passive appliances, the digital workflow simplifies fabrication, shortens delivery time, and allows rapid reproduction without repeat impressions. These features reduce procedural complexity and improve accessibility, particularly in centers with limited technical or laboratory resources. Findings should be interpreted cautiously as preliminary observations in an exploratory study. The feasibility demonstrated here provides a foundation for future developments such as smartphone-based intraoral scanning and AI-driven modules for direct digital impression capture using mobile devices.

## Supplemental Material

sj-docx-1-cpc-10.1177_10556656251400877 - Supplemental material for Smartphone and AI Workflow for 3D Printed Plate for Presurgical Therapy in Cleft Lip and Palate: Retrospective Evaluation of Outcomes Supplemental material, sj-docx-1-cpc-10.1177_10556656251400877 for Smartphone and AI Workflow for 3D Printed Plate for Presurgical Therapy in Cleft Lip and Palate: Retrospective Evaluation of Outcomes by Prasad Nalabothu, Hemwati Nandan, Srinivas Gosla Reddy and Andreas A. Mueller in The Cleft Palate Craniofacial Journal
