# Supplementary material for: Smartphone and AI Workflow for 3D Printed Plate for Presurgical Therapy in Cleft Lip and Palate: Retrospective Evaluation of Outcomes
Source: Cleft Palate Craniofac J. 2025 Nov 28;63(3):376–82. doi: 10.1177/10556656251400877 (PMC12953659; doi:10.1177/10556656251400877)
Supplement: sj-docx-1-cpc-10.1177_10556656251400877 - Supplemental material for Smartphone and AI Workflow for 3D Printed Plate for Presurgical Therapy in Cleft Lip and Palate: Retrospective Evaluation of Outcomes [file sj-docx-1-cpc-10.1177_10556656251400877.docx]

| Patient | Gender | Cleft side | Age at  first visit  (days)* | Duration of  plate therapy (days)** | Number of visits § | Initial fitting issues | Plate retention | Plate replacements |
| --- | --- | --- | --- | --- | --- | --- | --- | --- |
| 1 | Female | Left | 15 | 120 | 5 | No | Good | No |
| 2 | Male | Left | 10 | 118 | 5 | No | Good | No |
| 3 | Male | Left | 15 | 134 | 7 | Trimming at labial  vestibule | Reprint after 2 months | Yes (1 time) |
| 4 | Female | Right | 5 | 101 | 5 | No | Good | No |
| 5 | Female | Right | 10 | 129 | 5 | No | Good | No |
| 6 | Female | Left | 10 | 130 | 6 | No | Good | No |
| 7 | Female | Left | 5 | 107 | 5 | No | Good | No |
| 8 | Male | Right | 2 | 94 | 4 | No | Good | No |
| 9 | Female | Right | 5 | 120 | 5 | No | Good | No |
| 10 | Female | Left | 15 | 127 | 5 | No | Good | No |
| 11 | Female | Left | 5 | 96 | 5 | No | Good | No |
| 12 | Female | Left | 10 | 130 | 5 | No | Good | No |
| 13 | Female | Left | 10 | 135 | 6 | Trimming at frenulum | Good | No |
| 14 | Male | Right | 7 | 93 | 4 | No | Good | No |
| 15 | Male | Left | 5 | 120 | 5 | No | Good | No |
| 16 | Female | Left | 20 | 101 | 5 | No | Good | No |
| 17 | Male | Left | 15 | 105 | 4 | No | Good | No |
| 18 | Female | Left | 17 | 90 | 4 | No | Good | No |
| 19 | Female | Left | 25 | 130 | 6 | Trimming at labial vestibule | New plate | Yes (1 time) |
| 20 | Male | Left | 10 | 90 | 4 | No | Good | No |

**Table 1**. Characteristics of patients and outcomes of presurgical plate therapy following smartphone scanning of impressions and following automated plates.

*Mean Age at start of treatment = 10.8 ±5.7 (days)

**The average duration of therapy = 113.5 ±15.6 (days)

§The average number of visits from the start to the end of the treatment = 5 visits

**Table 2.** Comparative analysis of palatal morphological changes in infants with complete unilateral cleft lip and palate who underwent plate therapy after birth to 3-4 months of age (mean duration 113.5 days)

| **Parameters** | **T1**  **Mean ± SD** | **T2**  **Mean ± SD** | **p-value** | **Percentage change from T1 (%)** |
| --- | --- | --- | --- | --- |
| P*-L* (mm) | 10.27 ± 2.36 | - 1. 2.24 | P < 0.0001 | -56.8 |
| True Cleft (mm^2^) | 174.23 ± 34.03 | 103.15 ± 33.78 | P < 0.0001 | -40.8 |
| IMT angle | 74.29 ± 7.11 | 83.44 ± 3.37 | P < 0.0001 | +12.3 |
| Greater Surface (mm^2^) | 248.43 ± 46.36 | 337.37 ± 49.19 | P < 0.0001 | +35.8 |
| Lesser Surface (mm^2^) | 241.28 ± 40.09 | 322.35 ± 62.81 | P < 0.0001 | +33.5 |
| Premaxilla Surface(mm^2^) | 100.18 ± 25.33 | 105.05 ± 22.8 | 0.14 | +4.9 |
| Total palate surface (mm^2^) ^†^ | 589.9 ± 100.3 | 764.79 ± 119.3 | P < 0.0001 | +29.6 |

P < 0.05 considered statistical significance

**^†^** (total palate surface: greater surface, lesser surface and premaxilla)
